# The natural history of cardiovascular risk factors in health professionals: 20-year follow-up

**DOI:** 10.1186/s12889-015-2477-8

**Published:** 2015-11-11

**Authors:** Thiago Veiga Jardim, Ana Luiza Lima Sousa, Thais Inacio Rolim Povoa, Weimar Kunz Sebba Barroso, Brunela Chinem, Luciana Jardim, Rafaela Bernardes, Antonio Coca, Paulo Cesar Brandão Veiga Jardim

**Affiliations:** Hypertension League, Federal University of Goiás, Primeira Avenida Sem número, Setor Universitário, CEP 74000-000 Goiânia Goiás, Brazil; Hypertension and Vascular Risk Unit, Hospital Clínic (IDIBAPS), University of Barcelona, Barcelona, Spain

## Abstract

**Background:**

The knowledge of the presence and evolution of cardiovascular risk factors in young people may significantly contribute to actions to modify the natural history of these risks and prevent the onset of cardiovascular disease.

**Objectives:**

To assess the presence and evolution of cardiovascular risk factors in health professionals over a 20-year period.

**Methods:**

A group of individuals was evaluated when they first started graduate programs in medicine, nursing, nutrition, dentistry, and pharmacy, and 20 years later. Data obtained in the two phases were compared. Questionnaires about hypertension, diabetes, hypercholesterolemia, family history of early-onset cardiovascular disease, smoking, alcohol consumption, and sedentary lifestyle were administered. Cholesterol, blood glucose, blood pressure, weight, height, and body mass index (BMI) were measured.

**Results:**

Of the 281 individuals (62.9 % women; mean age 19.7 years) initially analyzed, 215 (59.07 % women; mean age 39.8 years) were analyzed 20 years later. An increase in mean values of systolic (111.6 vs 118.7 mmHg– *p* < 0.001) and diastolic blood pressure (71vs 77.1 mmHg – *p* < 0.001), cholesterol (150.1 vs 182.4 mg/dL – *p* < 0.001), blood glucose (74.3 vs 81.4 mg/dL – *p* < 0.001) and BMI (20.7 vs 23.7 kg/m^2^ – *p* = 0.017) was observed. Despite the decrease of sedentarism (50.2 vs 38.1 % - *p* = 0.015), the prevalence of hypertension (4.6 vs 18.6 % - *p* < 0.001), excessive weight (8.2 vs 32.1 % - *p* < 0.001), hypercholesterolemia (7.8 vs 24.2 % - *p* < 0.001), and alcohol consumption (32.7 vs 34.9 % - *p* = 0.037) increased. There was no change in the prevalence of smoking.

**Conclusion:**

Health professionals presented an increase in systolic and diastolic blood pressure, blood glucose, body mass index, and cholesterol over the 20-year study period. Regarding the prevalence of cardiovascular risk factors, increased blood pressure, overweight, hypercholesterolemia and alcohol consumption, and a decrease in sedentary lifestyle were observed.

## Background

Cardiovascular diseases (CVD) are the leading cause of death worldwide. In 2008, approximately 17.3 million people died of CVD, accounting for approximately 30 % of all deaths globally. Of these deaths, an estimated 7.3 million were caused by coronary heart disease (CHD) and 6.2 million by stroke [1]. It is projected that deaths due to CVD, mainly from heart disease and stroke, will increase to approximately 23.3 million by 2030, and CVD will remain the leading cause of death worldwide [2].

An unhealthy diet, sedentary lifestyle, smoking, and excessive alcohol consumption are the most important behavioral risk factors and are responsible for approximately 80 % of cases of CHD and cerebrovascular disease [1].

The effects of an unhealthy diet and a sedentary lifestyle can be identified via high blood pressure and high levels of blood glucose and lipids, in addition to overweight and obesity. These risk factors can be identified in primary healthcare services and indicate an increased chance of acute myocardial infarction, stroke, heart failure, and other complications [3].

Knowledge of cardiovascular risk factors (CVRF) and the presence of these factors in young people, their evolution over time, and an assessment of behavioral risk factors may significantly contribute to actions that may modify the natural history of these risks and, therefore, prevent the onset of CVD [4–6].

There are no follow up studies adressing CVRF evolution in young populations whith health-related degrees, investigating the possible effect of health education as a preventive tool for the onset of these risk factors and as a consequence CVD.

Therefore, we analyzed the presence and evolution of CVRF over a 20-year period in a population of individuals who, initially, were undergraduate students in a health-related field (medicine, nursing, nutrition, dentistry, or pharmacy) and were professionals working in these fields at the end of the study.

## Methods

This was a longitudinal study in which evaluations were performed on two occasions, 20 years apart. The study population was composed of undergraduate students from the Schools of Medicine, Nursing, Nutrition, Dentistry, and Pharmacy of the Federal University of Goiás (UFG), which is located in a large city in the Midwest region of Brazil. The first contact with the students occurred during an interval in their regular activities, and all the students who started their degree programs in 1993 were invited to attend the study by the investigators. The same individuals were reassessed 20 years later, when they were working in health-related professions.

Individuals who declined to participate in any phase of the study and patients with congenital heart disease and type 1 diabetes were excluded. In the first phase, study subjects data colection was performed on pre-scheduled dates in accordance with the University Administration. In the second phase, subjects were located through the regional state councils of medicine, nursing, nutrition, dentistry, and pharmacy and then contacted by telephone to schedule an in-person interview for data collection. Individuals were also located by name, using the Internet as a search tool. Individuals who did not live in the region were interviewed by telephone. Because the subjects were health professionals, the reported data were included in the final analysis. All participants were informed about the study procedures in 1993 and signed an informed consent form. Informed consent was collected again for the second phase of the study.

The questionnaire used in 1993 was applied again 20 years later. The variables analyzed in the two phases of the study were age, gender, and diagnosis or previous treatment of hypertension, hypercholesterolemia, or diabetes. Study subjects were also asked about the occurrence of major cardiovascular events (acute myocardial infarction [AMI], stroke, or need for myocardial revascularization [MRV]). Questions on lifestyle factors included the history of smoking (smoker or non-smoker), alcohol consumption (drinking alcohol or not) and physical activity (a - sedentary: no physical activity; irregular physical activity; physical activity for < 30 min three times/week; b - regular physical activity: physical activity for ≥ 30 min at least three times/week). The presence of early-onset cardiovascular disease in first-degree relatives (<65 years for women and <55 years for men) was also analysed.

### Objective measures

The following parameters were evaluated:

- Weight: Individuals were weighed wearing light clothing and without shoes using a digital Plena Lithium scale with a maximum capacity of 150 kg and an accuracy of 100 g.
- Height: Individuals without shoes were measured using a Seca laser stadiometer, model 206, with an accuracy of 0.1 cm.
- Body mass index (BMI): BMI was calculated using the formula: (BMI = weight in kg/height^2^ in meters) [7]. Values were categorized using World Health Organization cut-off points, as follows: underweight < 18.5 kg/m^2^; normal 18.5–24.9 kg/m^2^; overweight 25–29.9 kg/m^2^; obesity grade I 30–34.9 kg/m^2^; obesity grade II 35–39.9 kg/m^2^; and obesity grade III 40 kg/m^2^ or more [8]. Values of more than 25 kg/m^2^ were categorized as excessive weight.

Blood pressure (BP): Blood pressure was measured with mercury column devices in the first phase of the study; correctly calibrated semiautomatic devices (Omron Hem-705 CP) were used in the second phase. After 5 min of rest, two measurements were taken at intervals of 2 min from the right arm, with the individual seated and his/her arm supported. The second blood pressure measurement was used for data analysis. The use of different devices to measure blood pressure was not considered relevant because a validated semi-automated device [9] and a standardized measurement technique were used in the two phases [10]. Hypertension was defined according to the 2013 ESH/ESC Guidelines for the management of arterial hypertension [10]: SBP > =140 mmHg and/or DBP > =90 mmHg.

### Data collection via telefone

Data collection via telephone was made in 16 individuals. In these cases, reported weights and heights were used, and individuals were asked to measure their BP with calibrated equipment used in daily practice, following the study recommendations. Because these individuals were health professionals, these procedures were performed without difficulties, and the data were considered reliable.

### Laboratory data

In the first phase of the study, blood glucose and cholesterol were measured after a 12-hour fast in blood samples taken via finger stick with a lancet; readings were performed via the tape method using the Hemoglucotest and Reflotron devices, respectively.

For the second phase of the study, fasting blood glucose tests and lipid profiles performed within 12 months before filling out the questionnaire were used. The samples were collected after a 12-hour fast, and individuals were instructed to not drink alcohol for 48 h before collection. The enzymatic colorimetric method was used to determine total cholesterol (TC), high-density lipoprotein (HDL) cholesterol, triglycerides (TG), and plasma glucose levels. Low-density lipoprotein (LDL) cholesterol values were estimated using the Friedewald equation, where LDL = TC–(HDL + TG/5) [11].

Although the methods used to measure cholesterol and blood glucose were not the same in the two study phases, the correlation between the values obtained by these two methods is well established, suggesting that the different methods had no impact on the data analysis [12–15].

### Database and statistical analysis

The data were organized into databases using Microsoft Excel and were comparatively analyzed. The Kolmogorov-Smirnov test was used to determine whether continuous variables were normally distributed. The Student’s *t*-test was used to compare numerical variables, expressed as the mean and standard deviation. A comparative analysis of CVRF between 1993 and 2013 was performed using McNemar’s test. The correlation of numerical variables between the two phases of the study was analyzed using Pearson’s correlation test. Values of *p* < 0.05 were considered statistically-significant. The statistical analysis was performed using SPSS software (Statistical Package for the Social Sciences, version 20.0, Chicago, IL, USA).

### Ethics

The research project was submitted to and approved by the Research Ethics Committee of the Clinic Hospital, Federal University of Goiás.

## Results

The database for the first phase of the study was composed of 281 individuals; 220 (78.3 %) of these were located 20 years later for the second phase. Five individuals were excluded: four did not agree to participate in the second phase of the study, and one was diagnosed with type 1 diabetes between the first and second phases. Therefore, 215 health professionals were included in the final analysis.

Of the 281 individuals studied in 1993, 62.99 % were female, and the mean age was 19.7 years (minimum 17 and maximum 22 years). In the 215 subjects studied in 2013, the mean age was 39.8 years (minimum 37 and maximum 42 years), and 59.07 % were female.

In the first phase of the study, no individual reported having hypertension, hypercholesterolemia, or diabetes. However in the second analysis, eight individuals reported having hypertension, all of whom were receiving treatment. The number of individuals with hypercholesterolemia was higher: 18 individuals reported having elevated cholesterol levels, 9 (50 %) of whom treatment (pharmacological or otherwise). No individual reported having type two diabetes.

There were no reports of early-onset cardiovascular events in first-degree relatives in 1993. However, this type of event was reported by 47 individuals in 2013.

No cardiovascular events had occurred in the 215 individuals included in the second analysis.

Analysis of the prevalence of CVRF in the 215 individuals included in both analyses showed a significant increase in hypertension, excessive weight, hypercholesterolemia, and alcohol consumption, and a reduction in the prevalence of sedentary lifestyles. For smoking, no significant change was observed (Table 1).

**Table 1:** Prevalence of different cardiovascular risk factors in 1993 and 2013 in the studied population. Goiania, Goiás State, Brazil

	1993 (*n* = 281)	2013 (*n* = 215)	*p* *
Hypercholesterolemia	7.8 % (22)	24.2 % (52)	<0.001
Hypertension	4.6 % (13)	18.6 % (40)	<0.001
BMI ≥ 25 kg/m^2^	8.2 % (23)	32.1 % (69)	<0.001
Sedentary lifestyle	50.2 % (141)	38.1 % (82)	0.015
Smoking	4.6 % (13)	3.7 % (8)	0.289
Alcohol consumption	32.7 % (92)	34.9 % (75)	0.037

*McNemar’s test, significant at p < 0.05. Values are expressed as percentages and (absolute numbers). *BMI* body mass index. Hypercholesterolemia – total cholesterol > 200 mg/dL and/or treatment; Hypertension – SBP > =140 mmHg and/or DBP > = 90 mmHg and/or treatment

Assessment of the prevalence of CVRF by gender showed an increased prevalence of hypercholesterolemia, hypertension, and excessive weight in men and women. The reduction in sedentary lifestyle was significant only in women, as was the increase in alcohol consumption. There was no difference in the prevalence of smoking according to gender. There was a significant increase in total mean values of systolic blood pressure (SBP), diastolic blood pressure (DBP), BMI, blood glucose, and cholesterol between the first and second study phases. (Table 2).

**Table 2:** Mean values of SBP, DBP, BMI, blood glucose and cholesterol in 1993 and 2013 in the studied population. Goiânia, Goiás State, Brazil

	1993 (*n* = 281)	2013 (*n* = 215)	*p* *
SBP (mmHg)	111.6 ± 13.2	118.7 ± 13.9	<0.001
DBP (mmHg)	71.0 ± 8.7	77.1 ± 9.2	<0.001
BMI (kg/m^2^)	20.7 ± 2.1	23.7 ± 3.9	0.017
Blood glucose (mg/dL)	74.3 ± 6.2	81.4 ± 8.6	<0.001
Cholesterol(mg/dL)	150.1 ± 32.9	182.4 ± 28.3	<0.001

Values expressed as mean ± standard deviation. *Student’s *t*-test for related samples, significant at *p* < 0.05. *SBP* systolic blood pressure; *DBP*; diastolic blood pressure; *BMI* body mass index

Assessment of these variables by gender, showed both men and women had a significant increase in all these parameters.

**Fig. 1:**
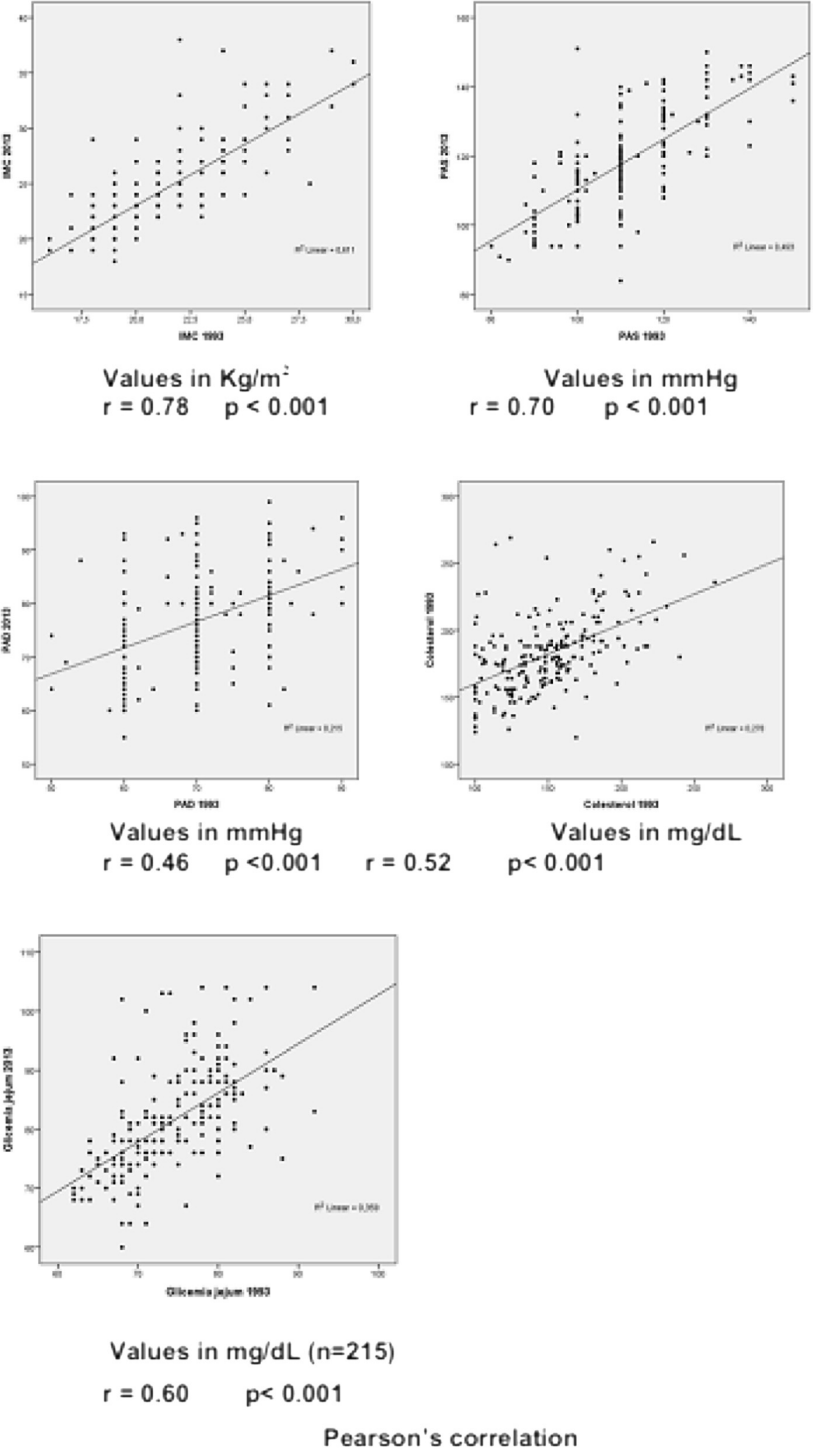
Correlation between body mass index (BMI), systolic blood pressure (SBP), diastolic blood pressure (DBP), cholesterol and blood glucose levels between 1993 and 2013 in the studied population (*n* = 215)

The analysis showed a positive correlation between BMI, SBP, DBP, cholesterol, and blood glucose levels obtained in 1993 with those found in 2013 (Fig. 1), demonstrating the early onset of risk factors and their maintenance over time.

## Discussion

This study aids understanding of the natural history of cardiovascular disease. Over a 20-year follow-up, a significant increase in the prevalence of some CVRF (excessive weight, hypercholesterolemia, hypertension, and alcohol consumption) was found despite a significant reduction in the prevalence of sedentary lifestyles. There was no significant change in tobacco smoking. These findings are very similar to the findings of an earlier study of the physicians included in the present study when analyzed 15 years after the first analysis [16], although the current study included a broader group of health professional and not only the physicians, over a 5 years longer period of time.

Health education is an essential tool in the prevention and control of chronic non-communicable diseases [17–19]. Monitoring the evolution of some risk factors for CVD in individuals with health-related degrees could, theoretically, determine the real impact of health education as a measure of health promotion because this population has formal training in healthcare and therefore has deeper knowledge of the risks and effects of risk factors for CVD and the risk behaviors associated with these diseases.

An increased prevalence of overweight over time has been reported by Brazilian and international studies analyzing the evolution of the BMI in specific populations [20–23]. Although the population examined in this study had received formal education in health-related fields, the evolution the BMI was similar to that of the general population, with a significant increase in the prevalence of excessive weight over a 20-year follow-up. However, when the prevalence of excessive weight in the study population was compared with data from individuals belonging to the same age group and living in Brazilian state capitals and the Federal District, the study population presented lower rates (32,1 × 55,9 %), even when the study population was compared only with the general population with higher education levels (>12 years of education) [24] (32,1 × 48,4 %).

Despite a significant rise in hypercholesterolemia over the study period, the prevalence was much lower in the study population than in the general population. A study in nine Brazilian state capitals in individuals with a mean age of 35.4 years found total cholesterol levels >200 mg/dL in 38 % of males and in 42 % of females [25], compared with 24.2 % of males and females in the study population. Another large study of more than 81,000 individuals with a mean age of approximately 43 years from 13 cities in different regions of Brazil also found a higher prevalence of hypercholesterolemia (40 %) [26] compared with the present study.

Comparing the number of hypertensive individuals in the study population in both evaluation periods with the population of a large city in the same region [27] divided by age group showed that study participants had lower rates of hypertension in both phases, even though there was an increase in the prevalence of hypertension between the first and second phases. These data corroborate the relationship between hypertension and low education levels [28] as the study population had a higher level of education than that of the general population of the city.

Alcohol consumption increased significantly in study subjects between the first and second study phases, and the prevalence was similar to that of the population of the Brazilian state capitals. There was a trend to an association between increased alcohol consumption and higher educational levels [28].

There was a significant reduction in sedentary lifestyles from 50.2 % in the first phase to 38.1 % in the second phase. In the general population of nine Brazilian capitals, the prevalence of sedentary lifestyles ranged from 28.2 to 54.5 %; however, unlike in the study subjects, the general Brazilian population tended to be more active between 15 and 24 years of age [28]. Our results showed an inverse relationship between educational level and the prevalence of sedentary lifestyles, similar to the results of a Brazilian telephone-based survey of risk and protective factors for chronic diseases (VIGITEL) [24].

We found that even among the CVRF that increased significantly in the study period, the prevalence rates were lower than those observed in the general population. These data are in agreement with findings from the Nurses Health Study II [29] and the Physicians Health Study I [30], in which the prevalence of CVRF was significantly-lower among American nurses and physicians, respectively, compared with the general population [31–33].

The VIGITEL study [24] showed a prevalence of smoking of 12.1 %. Smoking was more prevalent in individuals with lower educational levels and in those aged between 25 and 65 years. The prevalence rates of smoking of 4.6 and 3.7 % found in the first and second phases of our study, respectively, show that the health professionals analyzed study smoke less than the general population. We also found a trend to an association between less smoking and higher educational levels, similar to Brazilian [24] and international [34] data.

We found a similar increase in excessive weight, hypercholesterolemia, and hypertension between men and women between the first and second study phases, but no difference in smoking. There was a significant reduction in sedentary lifestyle, and an increase in alcohol consumption in women but not in men. The results on sedentary lifestyle diverge from international [35] and Brazilian [24] data, which show higher rates of sedentary behavior among older women. The trend to increased alcohol consumption in women as they age is also not in agreement with other reports [24, 36].

Analyses of BP, cholesterol, blood glucose, and BMI values showed a significant increase in all of these variables over 20 years. The values of DBP, SBP, blood glucose, cholesterol, and BMI found in 1993 correlated with those obtained in 2013. Thus, individuals with higher levels in 1993 also had higher levels in 2013. These data may be useful for strategies for the early detection of modifiable risk factors and effective modifying interventions, as suggested by a study of CVRF in schoolchildren [37] and a report by the Bogalusa Heart Study [38].

One limitation of the study is that not all individuals assessed in 1993 were located in 2013. This may be due to the long time interval, the lack of updated and integrated records from Brazilian health regulators, and the mobility of the study population, with professionals working in regions distant from the study region. This latter factor is especially important because Brazil is a country of continental dimensions, with vast differences in regional development. Even so, the reassessment of > 75 % of the initial group makes the sample representative and supports the conclusions presented.

Another limitation was the use of different methods for cholesterol and blood glucose analysis in the two study phases. However, various reports confirm the correlation between the values obtained using these different methods, suggesting they have no impact on data analysis [12–15]. Likewise, the use of different devices to measure BP was not considered relevant because a validated semi-automated device [9] and a standardized measure technique [10] were used in both phases of the study.

Additional studies are needed to determine the possible impact of higher education on health care as a protective factor against chronic degenerative diseases, specifically CVD. This was not an initial goal of this study, which only aimed to assess the evolution of some CVRF over time. A comparison with reported data [24, 28] adjusted for age and educational level indicates a positive difference in health professionals compared with the general population; however, only a study with an appropriate design could objectively answer this question. On the other hand, despite the decrease in sedentary lifestyle in this population there was an increase in the prevalence of hypertension, hypercholesterolemia and excessive weight, as well as an increase of the mean values of all the studied variables, suggesting that aging itself might be the most important aspect rellated to the onset of cardiovascular risk factors.

## Conclusions

In a longitudinal cohort of health professionals, increased SBP, DBP, blood glucose, BMI, and cholesterol were observed over a 20-year period. There was an increased prevalence of CVRF including hypertension, excessive weight, dyslipidemia, and alcohol consumption and a reduction in sedentary lifestyles.
